# Integrated single-cell and bulk characterization of branched chain amino acid metabolism-related key gene BCAT1 and association with prognosis and immunogenicity of clear cell renal cell carcinoma

**DOI:** 10.18632/aging.205506

**Published:** 2024-02-02

**Authors:** Jie Zheng, Yingqing Liu, Jiawei Wang, Jiewu Shi, Lin Li, Xuefeng Jiang, Lingsong Tao

**Affiliations:** 1 Department of Urology, Wuhu Hospital Affiliated to East China Normal University, Wuhu 241000, Anhui, People’s Republic of China

**Keywords:** branched chain amino acid, clear cell renal cell carcinoma, single-cell analysis, tumor microenvironment, BCAT1

## Abstract

Background: The relationship between clear cell renal cell carcinoma (ccRCC) and branched-chain amino acids (BCAA) metabolism has yet to be thoroughly explored.

Methods: The BCAA metabolism-related clusters were constructed using non-negative matrix factorization (NMF). The features of BCAA metabolism in ccRCC were evaluated by building a prognostic model using least absolute shrinkage and selection operator (LASSO) regression algorithm. Real-time quantitative PCR (RT-qPCR) was employed to analyze differential expression of branched-chain amino acid transaminase 1 (BCAT1) between cancer and paracancer tissues and between different cell lines. Cell counting kit-8, wound healing and Transwell chamber assays were conducted to determine changes in proliferative and metastatic abilities of A498 and 786-O cells.

Results: Two BCAA metabolism-related clusters with distinct prognostic and immune infiltration characteristics were identified in ccRCC. The BCAA metabolic signature (BMS) was capable of distinguishing immune features, tumor mutation burden, responses to immunotherapy, and drug sensitivity among ccRCC patients. RT-qPCR revealed overexpression of BCAT1 in ccRCC tissues and cell lines. Additionally, single-gene RNA sequencing analysis demonstrated significant enrichment of BCAT1 in macrophages and tumor cells. BCAT1 played tumor-promoting role in ccRCC and was closely associated with immunosuppressive cells and checkpoints. BCAT1 promoted ccRCC cell proliferation and metastasis.

Conclusions: The BMS played a crucial role in determining the prognosis, tumor mutation burden, responses to immunotherapy and drug sensitivity of ccRCC patients, as well as the immune cell infiltration features. BCAT1 was linked to immunosuppressive microenvironments and may offer new sights into ccRCC immunotherapeutic targets.

## INTRODUCTION

Renal cell carcinoma (RCC) constitutes 2% of all cancer cases globally, and its incidence is on a steady rise [1]. RCC comprises various histological subtypes, each presenting a unique set of pathological characteristics. Clear cell renal cell carcinoma (ccRCC), the most prevalent subtype, accounting for approximately 75% of all RCC cases, originates from the proximal tubular cells of the nephron [2]. Early resection is deemed to be most beneficial for ccRCC patients [3]; however, nearly 30% experience recurrence or metastasis post-tumor resection [4]. While anti-angiogenic therapies like Sunitinib and Pazopanib prove to be effective for metastatic RCC, resistance and relapse are common issues, with some patients showing intrinsic resistance to targeted therapies [5–7]. Identifying early prognostic biomarkers is essential for optimizing ccRCC treatment.

In situations with nutritional constraints, tumor cells exhibit aberrant proliferation, largely driven by metabolic reprogramming [8, 9]. Meeting the high demand for amino acids is crucial for sustaining tumor cell proliferation and growth [10, 11]. Branched-chain amino acids (BCAA), including leucine, isoleucine, and valine, are essential amino acids obtained solely from the diet [12]. Previous studies link the growth and metastasis of many malignancies to BCAA metabolism [13]. BCAA decomposition provides a carbon source for synthesizing molecules, promoting tricarboxylic acid cycle metabolism, oxidative phosphorylation, and energy supply for tumor cells. Additionally, BCAA decomposition products contribute nitrogen for de novo synthesis of nucleotides and amino acids. BCAA not only serves as protein-derived amino acids but also regulates protein synthesis through signaling, impacting the nutritional status [14, 15]. Despite the significant effects of BCAA metabolism on tumor progression, there is a lack of studies analyzing the specific mechanism of BCAA decomposition and uptake genes in ccRCC.

Branched-chain amino acid transaminase 1 (BCAT1), a key gene initiating BCAA catabolism located in the cytoplasm, transfers α-amino groups from BCAAs to α-ketoglutarate, converting BCAAs to the corresponding branched α-ketoacids and generating glutamate [16]. The study of BCAT1’s role in malignancies is intricate due to its tight association with various tumors. In acute myeloid leukemia, BCAT1 overexpression promotes cancer stem cell proliferation by regulating amino acid metabolism [17]. High BCAT1 expression also drives metastasis and proliferation in hepatocellular carcinoma through activating epithelial-mesenchymal transition (EMT) [18]. However, BCAT1 inhibition doesn’t uniformly suppress tumor growth, and its ability to promote progression and metastasis isn’t universally acknowledged. For instance, BCAT1 inhibition in pancreatic ductal adenocarcinoma (PDAC) didn’t inhibit tumor growth, with lower BCAT1 expression in tumors and higher BCAA levels in plasma [19]. These studies suggest that BCAT1 is intricately involved in cancer progression, but its prognostic value and biological mechanism in ccRCC remain unclear.

This study identified two BCAA metabolism-related clusters with distinct prognostic and immune features based on the expression profiles of BCAA metabolism-related genes (BMGs). The BCAA metabolic signature (BMS) was constructed and validated to predict ccRCC patient prognosis using data from public databases. We analyzed the model’s correlation with mutation, immune invasion, immunotherapy, tumor microenvironment, and drug sensitivity. Additionally, we explored the clinical characteristics, biological pathways, and features of BCAT1, a crucial gene in BCAA metabolism.

## MATERIALS AND METHODS

### Data acquisition and processing

Transcriptomic data and clinical information for ccRCC were sourced from The Cancer Genome Atlas (TCGA) and Gene Expression Omnibus (GEO) databases. Eighteen BMGs were compiled from pertinent reviews and studies [12, 19, 20]. Datasets E-MTAB-1980 and GSE22541 were used for objective evaluation, while GSE17895, GSE40435, GSE53737, and GSE73731 were employed for validating the association of clinicopathological characteristics with BCAT1 expression. The Clinical Proteomic Tumor Analysis Consortium (CPTAC) database provided protein expression data of candidate genes. Real-time quantitative PCR (RT-qPCR) assessed BCAT1 expression in ccRCC, the primers applied for BCAT1 were displayed as follows: “Forward: TGGCAAAACGTCTTCAGGAGG; Reverse: AGCTTGACTTAGTGGCTTTGG”. The primers applied for actin were displayed as follows: “Forward: AGCGAGCATCCCCCAAAGTT; Reverse: GGGCACGAAGGCTCATCATT”.

The GSE131685, GSE152938, and GSE171306 provided the single-cell sequencing datasets. They contained 4 ccRCC and 4 normal samples, totaling 64926 cells. Single-cell data were analyzed using the R package Seurat. The precise procedure was to first eliminate low-quality cells based on the criteria that the percentage of mitochondrial gene expression was less than 20, and that the number of expressed genes was greater than 100 but fewer than 6000. Additionally, we eliminate low-expressed genes based on the requirement that they be expressed in at least 100 cells. In the end, we had 50201 cells and 17304 genes. Then, to integrate the data and eliminate the batch effect, the FindIntegrationAnchors (where the reduction parameter was set to “rpca”) and IntegrateData function were conducted. To reduce the data’s dimensionality, the functions RunPCA and RunUMAP were conducted. The top 30 principal components and the top 2000 highly variable genes were utilized throughout. Finally, we set the resolution parameter to 1.5 and used the FindNeighbors and FindClusters functions to cluster and group the cells. We defined cell subpopulations using classical marker genes collected from the literature [21, 22]. The criteria of differential analysis in singe-cell analysis were set as adj.P-value< 0.001 and |log fold charge (FC)|> 1.

### Construction of BCAA metabolism-related clusters and bioinformatics analysis

Univariate cox regression analysis identified BMGs associated with prognosis. Then, based on the expression profiles of significant BMGs analyzed by univariate cox analysis, BCAA metabolism-related clusters were established using the non-negative matrix factorization (NMF) algorithm. Kaplan-Meier (KM) survival analysis determined the difference in overall survival (OS) between clusters. Differentially expressed genes (DEGs) were selected based on |log FC|> 2 and adj.P-value< 0.001. Gene Ontology (GO) functional enrichment and Kyoto Encyclopedia of Genes and Genomes (KEGG) pathway analyses were conducted on DEGs. Gene Set Variation Analysis (GSVA) enrichment analysis was performed using the R package GSVA and the gene set “c2.cp.kegg.v7.4.symbols” from MSigDB.

### Establishment of BCAA metabolic prognostic signature

Significant BMGs from univariate cox analysis underwent least absolute shrinkage and selector operation (LASSO) analysis to construct a prognostic gene signature. The BMS formula was derived through linear combination of gene expression weighted regression coefficients. Patients were stratified into high- and low-BMS groups based on the median BMS. Time-dependent receiver operating characteristic (ROC) curve and KM survival curve assessed BMS prognostic accuracy. Univariate and multivariate cox regression analyses evaluated the independence of BMS from other clinical phenotypes. Datasets E-MTAB-1980 and GSE22541 served as an objective evaluation cohort for BMS accuracy and stability.

### Evaluation of the immunogenomic landscape

Mainstream algorithms assessed immune infiltration scores, ssGSEA package gauged immune functional pathway enrichment, and Estimation of Stromal and Immune cells in Malignant Tumor tissues using Expression data (ESTIMATE) analysis quantified the immune scenario in the tumor microenvironment (TME). The tracking tumor immunophenotype (TIP, http://biocc.hrbmu.edu.cn/TIP/) tool evaluated standardized immune activity scores during the cancer immune cycle. Sensitivity to PD-1 and CTLA4 inhibitors was analyzed using immunotherapy sensitivity data from the Cancer Immunochromatography Database (TCIA, https://tcia.at/).

### Mutation and drug sensitivity analysis

The R package “maftools” analyzed somatic mutations, and tumor mutational burden (TMB) expression differences were assessed. KM curve evaluated survival differences between mutation and BMS combination. Six commonly used medications in ccRCC were evaluated for their half maximum inhibitory concentration (IC50) using the R package “pRRophetic” in the targeted treatment drug analysis.

### Western blot assay

Total proteins were extracted from the RCC cell lines, WB assay was performed after the detection of protein concentration. 20 μg of samples were separated on a 10% SDS-PAGE gel, then transferred to a PVDF membrane and blocked for 1 hour at room temperature. The membranes were incubated with primary antibodies (BCAT1 concentration, 0.5 μg/mL; GAPDH dilution rate, 1:500; Abcam, UK) at 4° C overnight. The next day, the membranes were incubated with the secondary antibody (Abcam; dilution rate, 1:2000) at 24° C for 1 h. Signals of targeted proteins were detected using an enhanced chemiluminescence detection system.

### Cell culture and cell transfection

Two human ccRCC cell lines (A498, 786-O) were purchased from the cell bank of the Chinese Academy of Sciences (Shanghai, China). All cells were cultured in RPMI 1640 medium (Thermo Fisher Scientific, Inc., USA) supplemented with 10% fetal bovine serum (FBS; Thermo Fisher Scientific, Inc.) at a constant temperature of 37° C in a humidified atmosphere containing 5% CO2.

Lentiviral shRNA plasmids that target BCAT1 together with the nonspecific control shRNA were obtained from Dharmacon (Shanghai, China). Transfection of plasmid and shRNA was performed with Lipo3000 following the manufacturer’s instructions.

### Cell counting kit-8 (CCK8) assay

Briefly, A498 and 786-O cells after different interventions were incubated in 96-well plates (2x10^3), supplemented with 200 μL culture medium and conditioned in 37° C with 5% CO2. On days 1, 2, 3, 4 and 5, 20 μL CCK-8 solution was added into each well, and incubation was performed for 2 h. Absorbance was measured at an optical density of 450 nm using a Microplate reader (Bio-Rad Laboratories, Inc., USA).

### Transwell assay

A498 and 786-O cells (with an incubation density of 2x10^5) were incubated in the upper chambers (Corning, USA). For the invasion assay, the upper chambers were pre-coated with Matrigel (BD Biosciences, USA). Culture medium without and with 10% FBS was added into the upper and lower chambers, respectively. After 12 h, non-migrated cells were wiped out while migrated or invaded CRC cells were fixed, stained and counted using an inverted microscope.

### Wound-healing assay

Cell migration was assessed by performing a wound healing assay. Briefly, A498 and 786-O cells were transfected with BCAT1. Approximately 2x10^6 cells were seeded into 6-well plates and cultured for 24 h. Then, a yellow plastic pipette tip was used to create a wound by scraping the cells. Cell migration was monitored under a Nicon Eclipse microscope and photographed at 100×.

## RESULTS

### Genetic variation prognoses of BMGs in ccRCC

To investigate the influence of BCAA on ccRCC progression, we analyzed mutation spectrum and copy number variations (CNVs) in BMGs across 336 samples. Only 21 samples (6.25%) exhibited mutations, with NOTCH2 having the highest mutation rate (Supplementary Figure 1A). CNV change analysis identified 18 genes with high frequency, predominantly showing copy number loss. MYC, NOTCH2, and SCL7A8 exhibited higher expansion increments, while ACADSB and NOTCH1 mainly experienced copy number loss (Supplementary Figure 1B). Differential mRNA expression analysis revealed 14 differentially expressed BMGs (P < 0.05), with 8 down-regulated and 6 up-regulated genes (Supplementary Figure 1C). Most differentially expressed BMGs exhibited prevalent CNVs (Supplementary Figure 1D). The comprehensive regulatory network illustrated interactions, associations, and prognostic value of the 18 BMGs (Supplementary Figure 1F).

### Prognostic and biological characteristics of BCAA metabolism-related clusters

**Figure 1:**
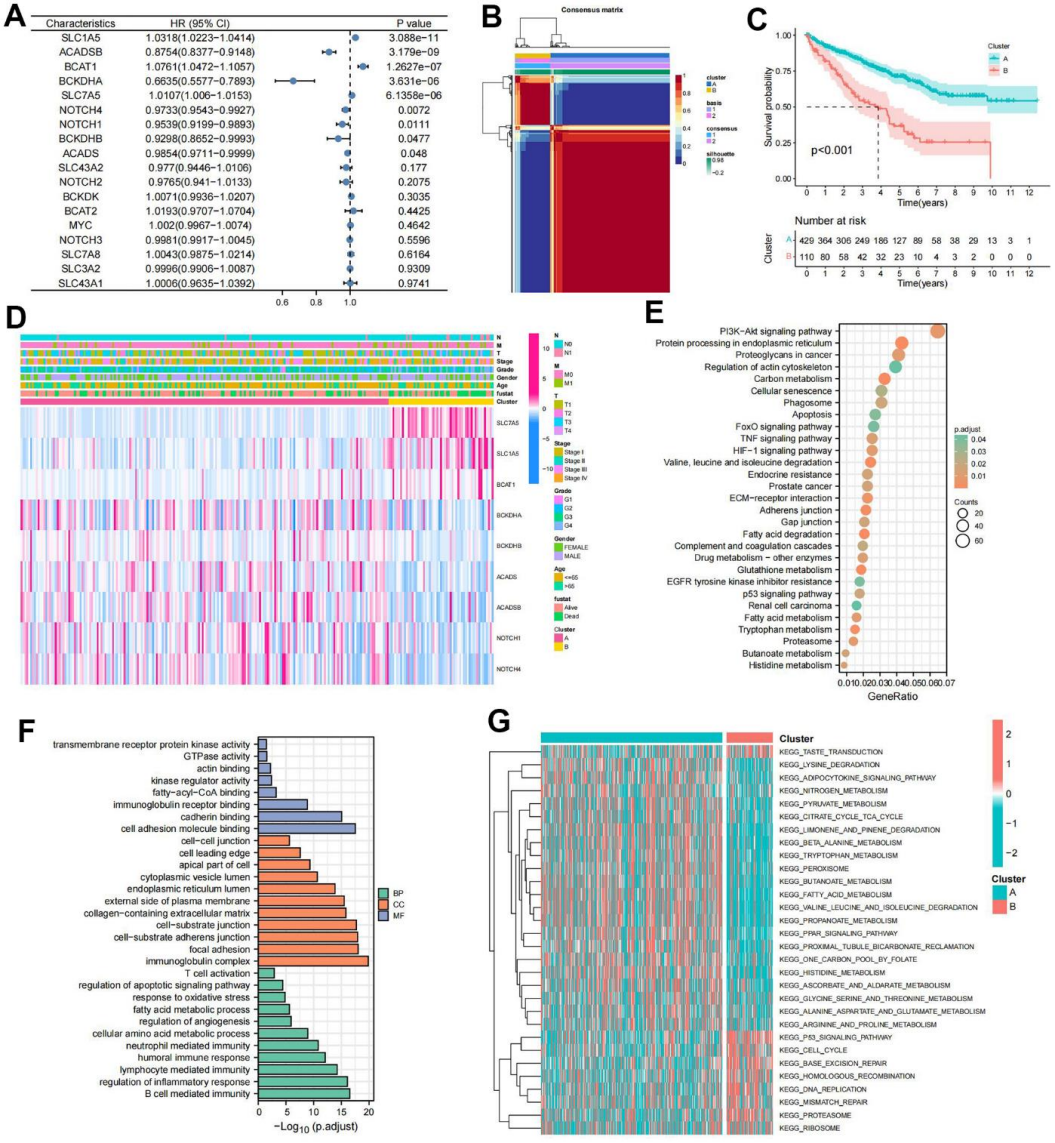
**Prognostic and biological characteristics of the branched chain amino acids (BCAA) metabolism-related clusters.** (**A**) Results of univariate cox analysis of BCAA metabolism-related genes. (**B**) Consensus map for non-negative matrix factorization (NMF) clustering. (**C**) The Kaplan-Meier (KM) survival curve of BCAA metabolism-related clusters. (**D**) Heatmap showing the correlations with clinicopathological characteristics based on results of the cluster analysis. (**E**) Kyoto encyclopedia of genes and genomes (KEGG) pathway enrichment of differentially expressed genes (DEGs) between clusters A and B. The enriched items were analyzed by using gene counts and adjusted p-values. (**F**) Gene ontology (GO) functional annotation analysis of DEGs between clusters A and B, including enriched biological processes (BP), cellular components (CC), and molecular functions (MF). (**G**) Results of Gene Set Variation Analysis (GSVA) enrichment analysis between clusters.

Univariate cox regression analysis of 18 BMGs guided the clustering of ccRCC patients into clusters A and B using the NMF algorithm (Figure 1A, 1B). Cluster B, associated with poor prognosis, exhibited significant survival differences compared to cluster A (Figure 1C). The heat map depicted BMGs expression profiles and clinical features (Figure 1D). DEGs between these clusters underwent GO and KEGG enrichment analyses performed. KEGG pathway analysis, revealing enrichment in renal cell carcinoma, oncogenic pathway, and metabolic pathway (Figure 1E). GO annotation classified proteins based on biological process (BP), cellular component (CC), and molecular function (MF), indicating involvement in multiple immune regulatory pathways, immunoglobulin complex, focal adhesion, and cell adhesion molecule binding, among others (Figure 1F). GSVA analysis indicated that multiple metabolic pathways including fatty acid metabolism, tryptophan metabolism, and beta-alanine metabolism were enriched in cluster A while multiple pro-cancer pathways including the P53 signaling pathway and cycle circle were enriched in cluster B (Figure 1G).

### Identification of immune characteristics of BCAA metabolism-related clusters

**Figure 2:**
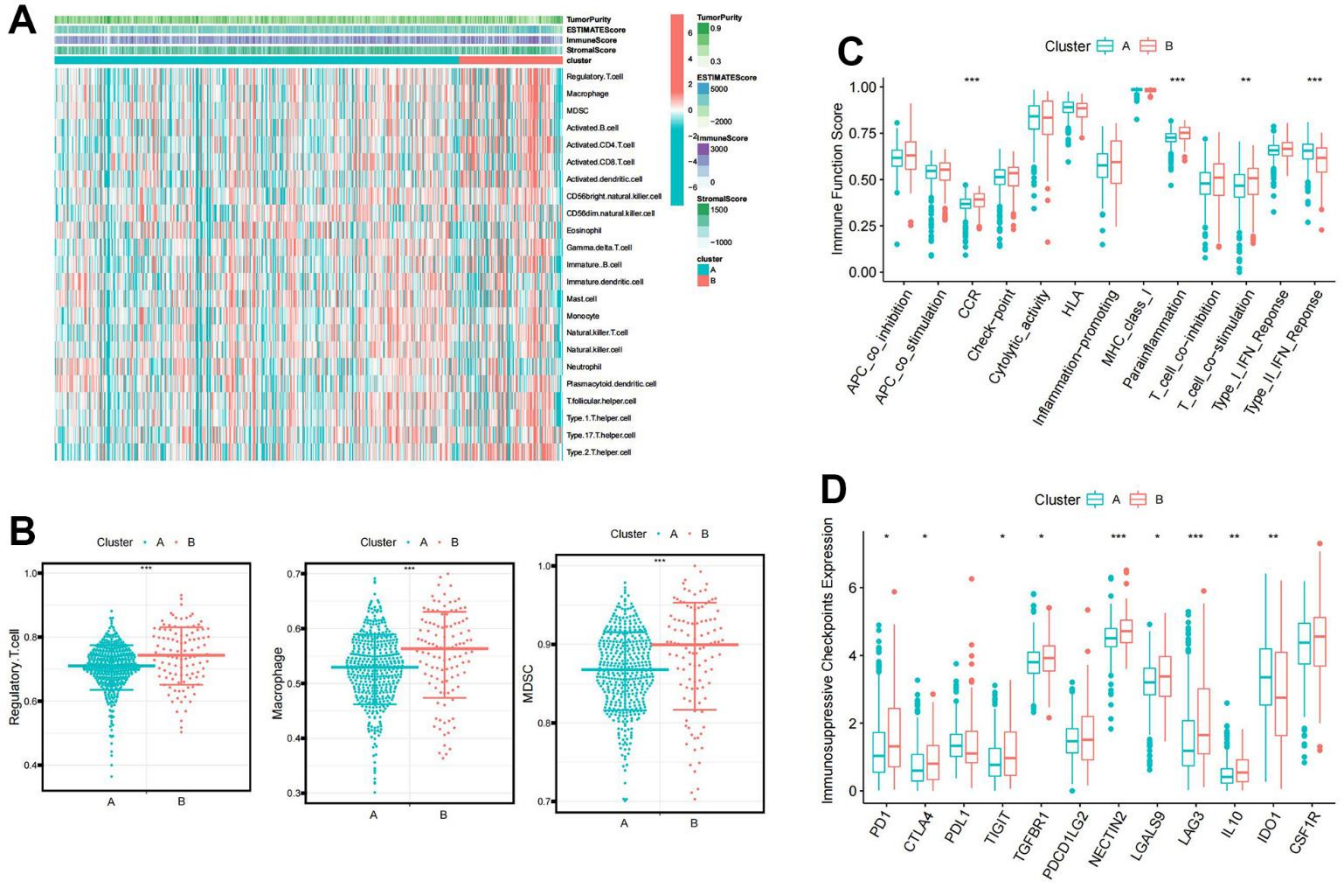
**The immune characteristic of the BCAA metabolism-related clusters.** (**A**) Distribution of immune infiltrating cells and tumor microenvironment scores between BCAA metabolism-related clusters. (**B**) Differential abundance of immunosuppressive cell infiltration (Macrophage, Regulatory.T.cell, and Myeloid-derived suppressor cells (MDSC)) between BCAA metabolism-related clusters. (**C**) Differences in the 13 immune-related functions between BCAA metabolism-related clusters. (**D**) Differential expression of immunosuppressive checkpoints between BCAA metabolism-related clusters. Wilcox test was used, and the asterisks represent the statistical *P*-value (*p <0.05, ** *p* <0.01, *** *p* <0.001).

A heat map visualized the distribution of immune infiltrating cells and tumor microenvironment scores between clusters A and B (Figure 2A). Immunosuppressive cells, such as Regulatory T cell, Macrophage, and Myeloid-derived suppressor cells (MDSC), were significantly more infiltrative in cluster B (Figure 2B). Immune function pathway indicated that higher expression of CCR, Parainflammation, T cell co-stimulation, and Type II IFN Response in cluster B (Figure 2C). Moreover, most immunosuppressive checkpoints were significantly overexpressed in cluster B compared to cluster A (Figure 2D).

### Construction of BCAA metabolic prognostic signature and BMS

**Figure 3:**
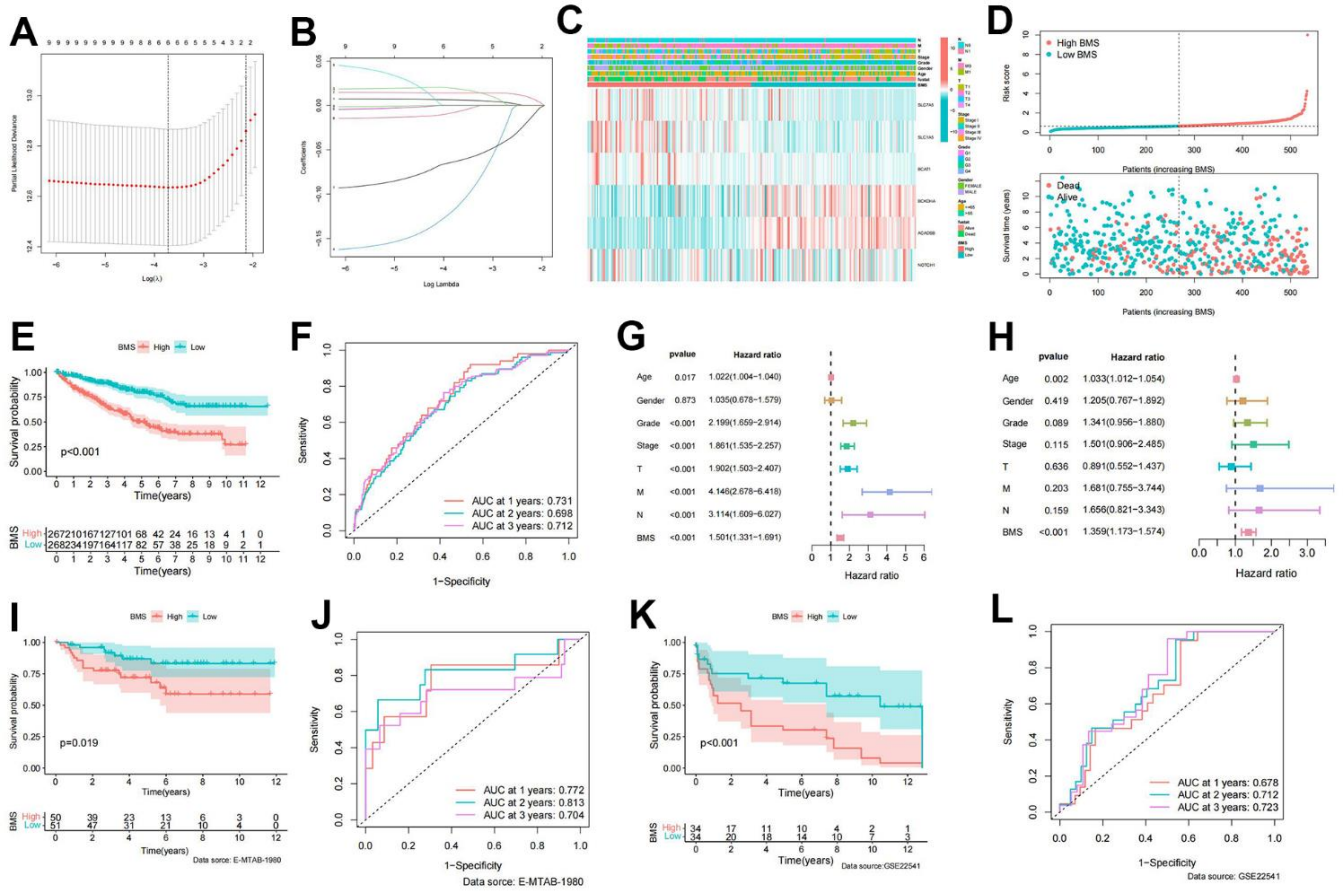
**Establishment of BCAA metabolism-related signature.** (**A**) LASSO coefficient profiles of the expression of 9 prognostic BCAA metabolism-related genes. (**B**) Selection of the penalty parameter (λ) in the least absolute shrinkage and selector operation (LASSO) model via 10-fold cross-validation. (**C**) Heatmap showing the model gene expression and clinicopathological variables in the BCAA metabolism-related signature (BMS); (**D**) Risk curve of the risk score rank, scatter plot for the survival status distribution; (**E**) The KM survival curve for the survival probability of patients in different groups; (**F**) Receiver operating characteristic (ROC) curve in predicting 1-,2-, and 3-year overall survival (OS) based on BMS. (**G**, **H**) Univariate and multivariate cox regression analyses of clinicopathological variables for predicting the survival in ccRCC patients. (**I**) The KM survival curve of BCAA metabolism-related signature in the E-MTAB-1980 cohort. (**J**) ROC curve in predicting 1-,2-, and 3-year OS based on BMS in the E-MTAB-1980 cohort. (**K**) The KM survival curve of BCAA metabolism-related signature in the GSE22541 cohort. (**L**) ROC curve in predicting 1-,2-, and 3-year OS based on BMS in the GSE22541 cohort.

LASSO regression analysis based on significant BMG expression profiles identified a BCAA metabolic prognostic signature comprising 6 BMGs (Figure 3A, 3B). The risk score of our signature can be figured out though the following formula:


$$\begin{array}{l} BMS=(-0.0070)*exp^{NOTCH1}+(-0.0626)\\ \;\;\;\;\;\;*exp^{ACADSB}+(-0.1140)\\ \;\;\;\;\;\;*exp^{BCKDHA}+(0.0188)*exp^{BCAT1}\\ \;\;\;\;\;\;+\;(0.0141)*exp^{SLC1A5}+(0.0061)\\ \;\;\;\;\;\;*exp^{SLC7A5} \end{array}$$

According to the median of BMS, ccRCC patients were equally split into high- and low-BMS groups. The heat map illustrated the distribution of 6 modeled gene expression profiles and clinicopathologic features (Figure 3C). Scatter plots and survival curves demonstrated higher BMS correlating with increasedc mortality (Figure 3D, 3E). The BMS displayed excellent accuracy in predicting 1-, 2-, and 3-year OS, with corresponding AUCs of 0.731, 0.698, and 0.712 (Figure 3F). Univariate and multivariate cox regression analyses identified BMS as the primary independent predictor of OS in ccRCC patients (Figure 3G, 3H). To further confirm the accuracy and stability of this signature, datasets from the E-MATB-1980 and GSE22541 were used as objective evaluation gene sets. The high-BMS group was linked with a poor prognosis, according to the KM survival curve, which showed a significant difference in survival between the high- and low-BMS groups (Figures 3I, 3K). Additionally, the E-MATB-1980 dataset’s AUCs for the 1-, 2-, and 3-year OS predictions based on BMS were 0.772, 0.813, and 0.70, respectively (Figure 3J). In the GSE22541 dataset, the AUCs for the 1-, 2-, and 3-year OS predictions based on BMS were 0.678, 0.712, and 0.723, respectively (Figure 3L).

### Identification of immune characteristic of BMS groups

**Figure 4:**
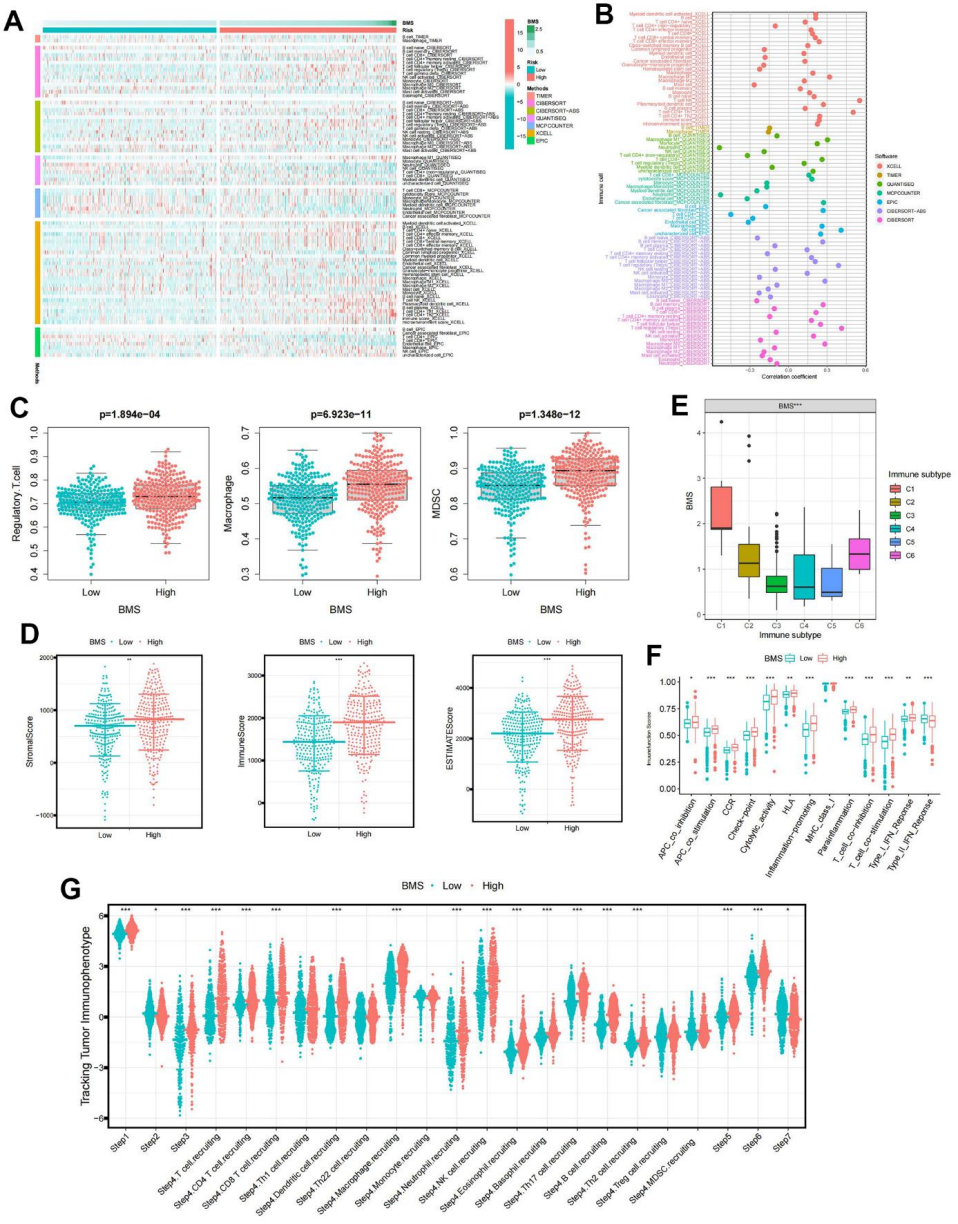
**The immune characteristic of BMS groups.** (**A**) Distribution of immune infiltrating cells between BMS groups using seven mainstream algorithms. (**B**) Correlation analysis of immune infiltrating cells between BMS groups using seven mainstream algorithms. (**C**) Abundances of main immunosuppressive infiltrating cells (MDSCs, macrophages, and regulatory T cells) in TME in two subgroups. (**D**) Samples in the high-BMS group exhibited higher ESTIMATE, stromal, and immune scores than the low-BMS group. (**E**) Association between BMS and the previously reported pan-cancer immune subtypes. (**F**) Differences in the 13 immune-related functions between high- and low-BMS groups. (**G**) Evaluation of the anticancer immunological functions of the seven-step cancer-immunity-cycle between high- and low- BMS groups. Wilcox test was used, and the asterisks represent the statistical *P*-value (*p <0.05, ** *p* <0.01, *** *p* <0.001).

We utilized seven mainstream algorithms to assess the immune infiltration scores in each ccRCC sample. The resulting heatmap illustrated the distribution of immune infiltration cells between high- and low-BMS groups (Figure 4A). Correlation analyses (Figure 4B) revealed a strong association between BMS and Macrophage and Regulatory.T.cell. Further examination of immunosuppressive cell expression indicated a significant increase in Regulatory.T.cell, Macrophage, and MDSCs abundance in the high-BMS group (Figure 4C). Using the ESTIMATE algorithm, we explored differences in immune-related scores between risk groups, observing higher ESTIMATE, Stromal, and Immune scores in high-BMS group (Figure 4D). To validate the reliability of BMS in immunotyping, we analyzed its association with pan-cancer immune subtypes, revealing higher BMS levels in ccRCC patients with C1 and C6 subtypes and lower levels in those with C3, C4, and C5 subtypes (Figure 4E) [23]. Previous studies have shown that C3 was related to a better prognosis while C6 was associated with a worse prognosis, which was consistent with our results. Additionally, the high-BMS group exhibited significantly elevated scores in all immune-related functions, except Type II IFN Response (Figure 4F). One previous study referred to seven sequential processes of antitumor immunity as the “cancer-immunity cycle” [24]. TIP (a web service for determining tumor immunophenotype profiling) was utilized to evaluate the anticancer immunological functions of the seven-step cancer-immunity-cycle between high- and low-BMS groups. Our results in Figure 4G revealed that the abundance of antitumor immune cells in high-BMS group was significantly higher than that in low-BMS group.

### Mutation and immunotherapeutic responses of BMS groups

**Figure 5:**
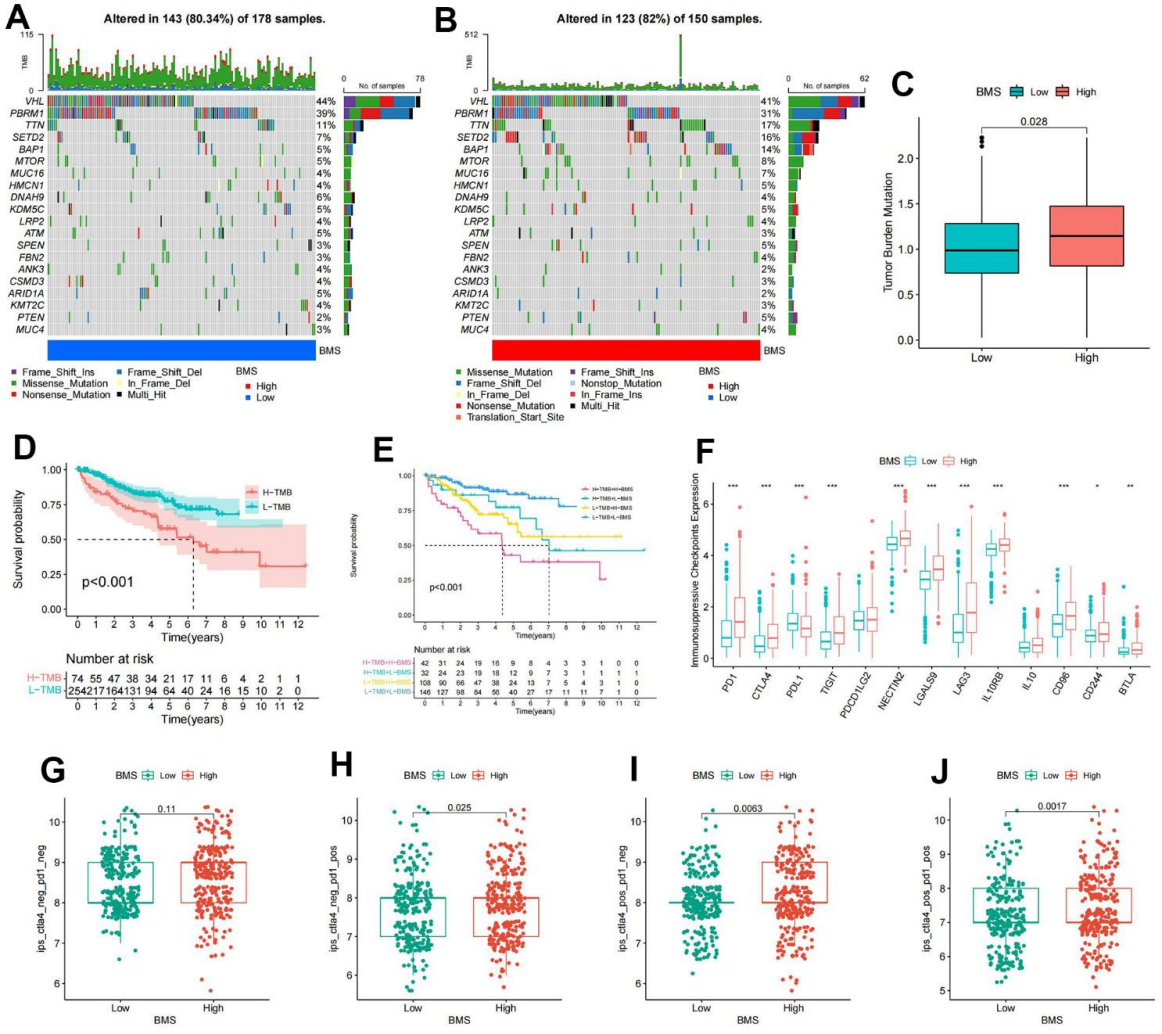
**Mutation and immunotherapeutic responses of BMS groups.** (**A**, **B**) Waterfall plots of somatic mutations in tumors in high-and low-BMS groups. (**C**) Differential expression of Tumor Mutation Burden (TMB) between high- and low-BMS groups. (**D**) Survival analysis between high- and low-TMB groups. (**E**) Survival analysis of distinct groups stratified by both TMB and BMS. (**F**) Differential expression of immunosuppressive checkpoints between high- and low-BMS groups. (**G**–**J**) The relative probability of response to anti-CTLA4 and/or anti-PD1 combination immunotherapy between high- and low-BMS groups. Wilcox test was used, and the asterisks represent the statistical *P*-value (*p <0.05, ** *p* <0.01, *** *p* <0.001).

Since TMB was substantially correlated with the effectiveness of immunotherapy, we analyzed the changes of TMB in high- and low-BMS groups. The mutation rate was 123/150 (82%) in the high-BMS group and 143/178 (80.34%) in the low-BMS group. The top 18 genes with the most significant mutations were all the same in the high- and low-BMS groups. (Figure 5A, 5B). As anticipated, the TMB was much higher in the high-BMS group compared to the low-BMS group (Figure 5C). Therefore, elevated TMB was considerably associated with poor prognosis (Figure 5D). Given the crucial link between TMB and immunotherapy efficacy, we analyzed TMB changes in high- and low-BMS groups. The mutation rate was higher in the high-BMS group, and the top 18 mutated genes were consistent between the groups (Figure 5A, 5B). As expected, TMB was significantly higher in the high-BMS group (Figure 5C), correlating with a poorer prognosis (Figure 5D). Combining TMB with BMS, we found distinct prognostic outcomes for different BMS and TMB combinations (Figure 5E). Analysis of immunosuppressive checkpoint expression revealed significant overexpression in the high-BMS group (Figure 5F). Considering a growing number of studies have demonstrated that high TMB was associated with better immunotherapy outcomes [23], we hypothesized that ccRCC patients with high BMS may benefit from immunotherapy despite owning a poorer prognosis. Hence, we downloaded immunotherapy data from the TCIA database to verify the value of BMS in predicting immunotherapy response, showing a higher probability of response to CTLA4(+)/PD-1(+), CTLA4(+)/PD-1(-), and CTLA4(-)/PD-1(+) treatments in the high-BMS group (Figure 5G, 5J).

### Drug susceptibility analysis of BMS groups

We compared the sensitivity of high- and low-BMS groups to commonly used ccRCC targeted therapeutic agents. The low-BMS group exhibited higher IC50 values for Rapamycin, Temsirolimus, Sorafenib, and Sunitinib, while Axitinib and Pazopanib showed no significant differences (Supplementary Figure 2A–2F). Target genes of anticancer drugs obtained from the DrugBank database included KIT, FLT1, FLT4, PDGFRA, RAF1, BRAF, RET, FGFR3, SH2B3, ITK, MTOR, FGF2, and FKBP1A. It was worth noting that Target gene expression analysis revealed significant differences in all target genes between the two groups (Supplementary Figure 3G), suggesting BMS as a potential tool for treatment selection.

### Clinical, prognostic, and protein expression characteristics of model genes

Analyzing model gene expression in different clinicopathological variables revealed strong correlations with clinicopathological stage. With the increase of the clinicopathological stage, the expression levels of ACADSB, BCKDHA, and NOTCH1 gradually decreased, while the expression levels of BCAT1, SLC7A5, and SLC1A5 gradually increased (Supplementary Figure 3A–3E). Supplementary Figure 3F revealed that BCAT1 and NOTCH1 were significantly overexpressed in ccRCC, while the expression of ACADSB and BCKDHA was significantly downregulated in ccRCC. There was no difference in the expression of SLC7A5 and SLC1A5 between the tumor and adjacent tissues. The protein expression data of the model genes were obtained from the CPTAC database and the differences in protein expression of the model genes between tumor and adjacent tissues were analyzed. ACADSB, BCKDHA, and SLC1A5 were less expressed in tumors, while BCAT1 and SLC7A5 were more expressed in tumors (Supplementary Figure 3G). The KM survival curves indicated that the expression levels of BCAT1, SLC7A5, and SLC1A5 were associated with a poorer prognosis, while the expression levels of ACADSB, BCKDHA, and NOTCH1 were correlated with a better prognosis (Supplementary Figure 3H). Therefore, considering the clinical, prognostic, and protein expression characteristics of the model genes, we finally screened out the oncogenic gene BCAT1 as a key gene for further analyses.

### Prognostic value and biological features of BCAT1

**Figure 6:**
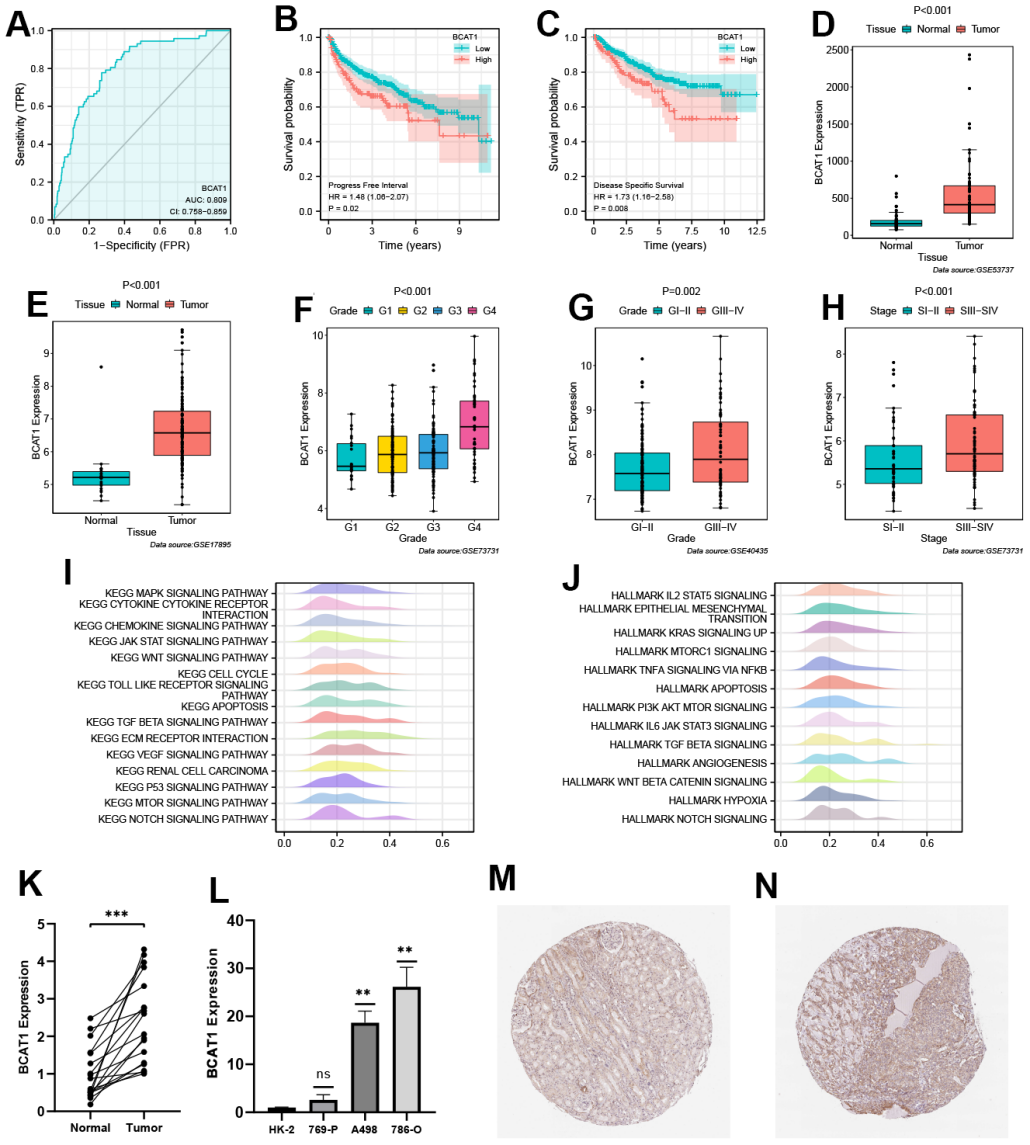
**Validation of prognostic value and biological features of branched-chain amino acid transaminase 1 (BCAT1).** (**A**) Time-dependent ROC curve of BCAT1 in ccRCC. (**B**, **C**) Kaplan-Meier survival curves of high- and low-BCAT groups in ccRCC (B: Progress Free Interval (PFI); C: Disease-Specific Survival (DSS)). (**D**–**H**) Validation of the correlation between BCAT1 expression and clinicopathological variables in ccRCC (D: GSE53737; E: GSE17895; F and H: GSE73731; G: GSE40435); (**I**) Results of Gene Set Enrichment Analysis (GSEA) analysis of BCAT1-enriched KEGG pathways. (**J**) Results of GSEA analysis of BCAT1-enriched HALLMARK pathways. (**K**) Comparison of mRNA expression levels of BCAT1 between ccRCC and adjacent cancerous tissues. (**L**) Comparison of mRNA expression levels of BCAT1 in HK-2 and RCC cell lines. (**M**, **N**) Immunohistochemistry (IHC) staining of BCAT1 in clinical ccRCC tissues (**N**) and adjacent cancerous tissues (**M**).

Patients were divided equally into high- and low-BCAT1 groups based on the median expression level of BCAT1 in ccRCC. BCAT1 exhibited strong predictive value in ccRCC patients (Figure 6A) and was associated with poor Progress Free Interval (PFI) and Disease-Specific Survival (DSS) (Figure 6B, 6C). Then, we utilized the GEO datasets to verify the correlation between BCAT1 expression and clinicopathological variables in ccRCC. BCAT1 expression was higher in ccRCC tissues, positively correlating with histological grade and pathological stage (Figure 6D–6H). To assess the biological features of BCAT1 in ccRCC, we performed the enrichment analyses of KEGG and hallmark pathways. In terms of KEGG pathways, overexpressed BCAT1 was positively associated with several tumor-related and angiogenesis-related pathways (Figure 6I), such as JAK/STAT signaling pathway, mTOR signaling pathway, VEGF signaling pathway, and TOLL like receptor pathway. And in terms of HALLMARK pathways, overexpressed BCAT1 was also positively associated with tumor-related and angiogenesis-related pathways (Figure 6J), such as KRAS signaling pathway, PI3K/Akt/mTOR signaling pathway, and Angiogenesis pathway. These results suggested that BCAT1 may play a potential driving role in tumorigenesis and progression.

### Validation of BCAT1 expression in ccRCC

RT-qPCR confirmed elevated BCAT1 expression in ccRCC tissues and all three ccRCC cell lines especially in 786-O cell (Figure 6K, 6L). Through Human Protein Atlas (HPA) database analysis, it was found that the protein expression level of BCAT1 in ccRCC tissues was significantly higher than that in adjacent tissues (Figure 6M, 6N).

### Identification of immune features of BCAT1

**Figure 7:**
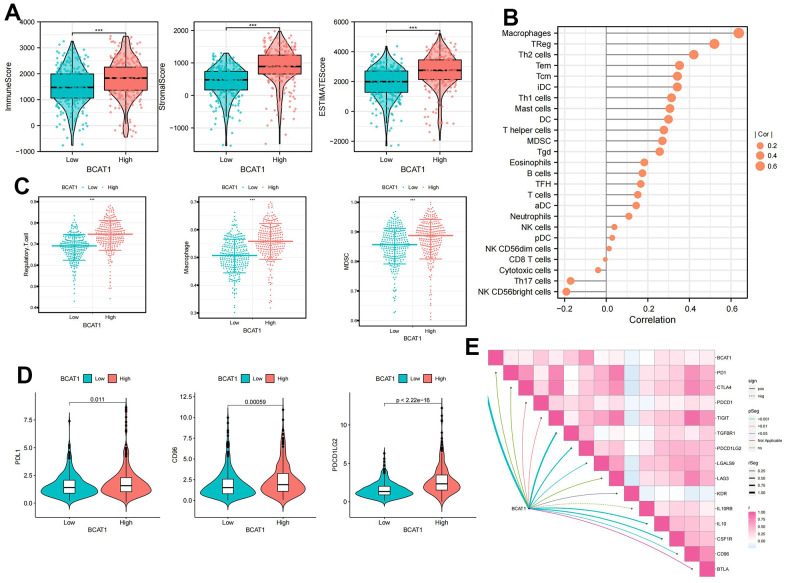
**Comparison of immune cells of ccRCC patients between high- and low-BCAT1 groups.** (**A**) Samples in the high-BCAT1 group exhibited higher ESTIMATE, stromal, and immune scores than the low-BCAT1 group. (**B**) Correlation analysis between BCAT1 expression and immune infiltrating cells. (**C**) Abundances of main immunosuppressive infiltrating cells (MDSCs, macrophages, and Tregs) in tumor microenvironment (TME) in two groups. (**D**) Differential expression of common immune checkpoint molecules (PDL1, CD96, and PDCD1LG2) between high- and low-BCAT1 groups. (**E**) Correlation between the expression of BCAT1 and common immune checkpoint molecules. Wilcox test was used, and the asterisks represent the statistical *P*-value (*p <0.05, ** *p* <0.01, *** *p* <0.001).

TME scores indicated that several immune cells and immune-related molecules were abundant in the high-BCAT1 group (Figure 7A). BCAT1 expression positively correlated with Regulatory T cells and Macrophages infiltration (Figure 7B), and high-BCAT1 group exhibited elevated immunosuppressive cell abundance (Figure 7C). Moreover, we investigated the relationship between BCAT1 and the immunosuppressive checkpoints. The results demonstrated that PDL1, CD96, and PDCD1LG2 were all over-expressed in the high-BCAT1 group, and BCAT1 was positively correlated with most immunosuppressive checkpoints (Figure 7D, 7E). Taken together, these data revealed that BCAT1 was closely related to the formation of an immunosuppressive microenvironment and may be a potential immunotherapeutic target.

### Mapping BCAT1 in single cell data

**Figure 8:**
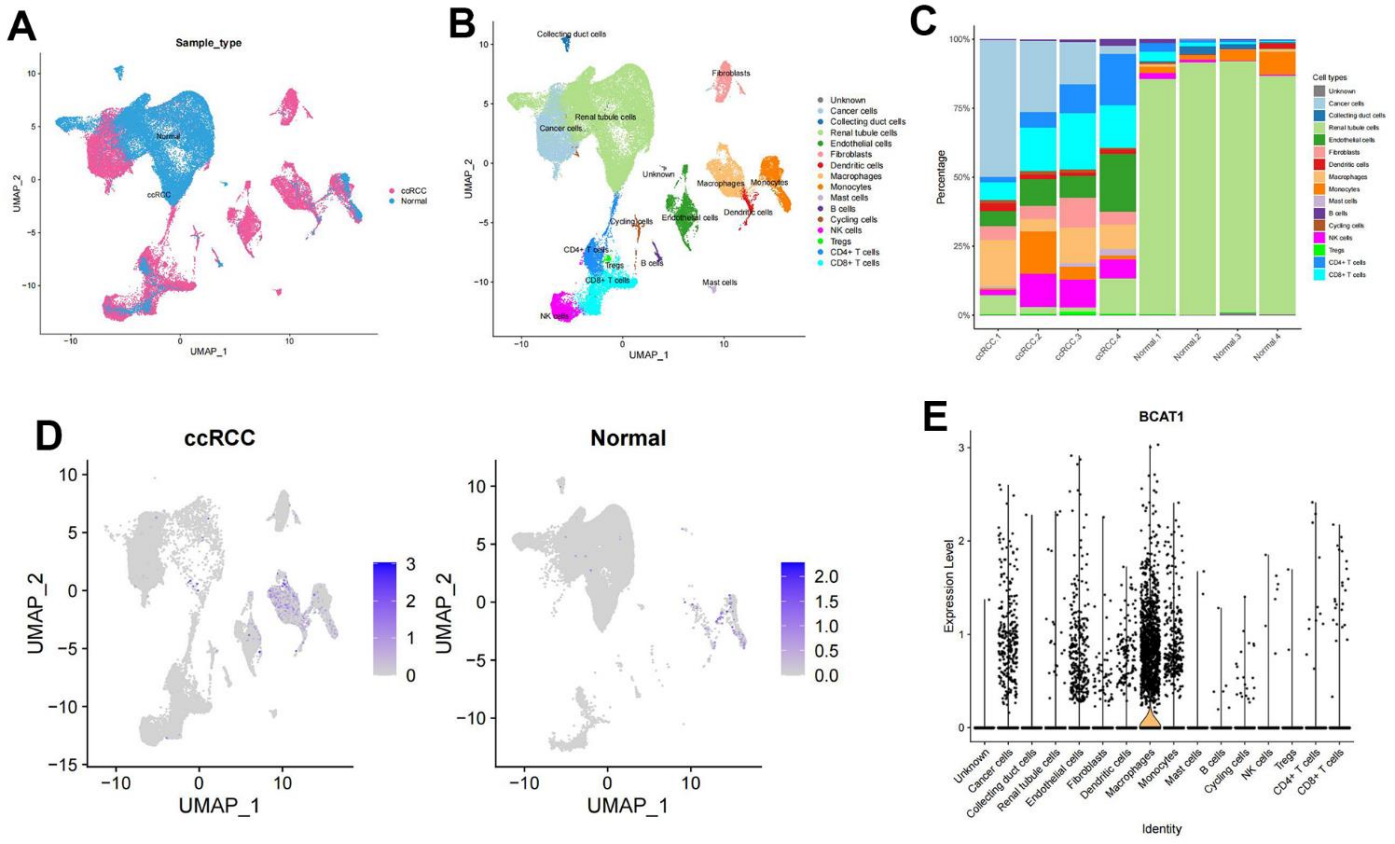
**Expression profile of BCAT1 based on single-cell sequencing analysis.** (**A**) Composition and distribution of single cells in the GEO datasets. (**B**) UMAP projection of all 50201 cells including ccRCC cells and normal kidney cells. 16 different cell clusters were identified. (**C**) Composition ratio of cell clusters in individual sample. (**D**) Distribution of the BCAT1 expression in scRNA-Seq cluster of ccRCC cells and normal cells. (**E**) Distribution of the BCAT1 expression of different cell clusters in ccRCC.

Figure 8A showed the composition and distribution of single cells from the GEO database, and 16 cell clusters were further identified (Figure 8B). In line with the earlier research [20], the majority of normal renal cortical samples were renal tubular epithelial cells, whereas immune and tumor cells predominated in ccRCC samples (Figure 8C). We then investigated the expression profile of BCAT1 in different types of cells. The results showed that the BCAT1 expression was significantly higher in macrophages and tumor cells than that in other cell types (Figure 8D, 8E), consistent with the former analysis. Thus, we can hypothesize that ccRCC may promote its own growth and progression through BCAT1-mediated metabolic reprogramming of BCAAs and induce the same process of macrophages in the TME, which leads to the polarization of immunosuppressive phenotype.

### BCAT1-knockdown suppressed proliferation, migration, and invasion in A498 and 786-O cells

**Figure 9:**
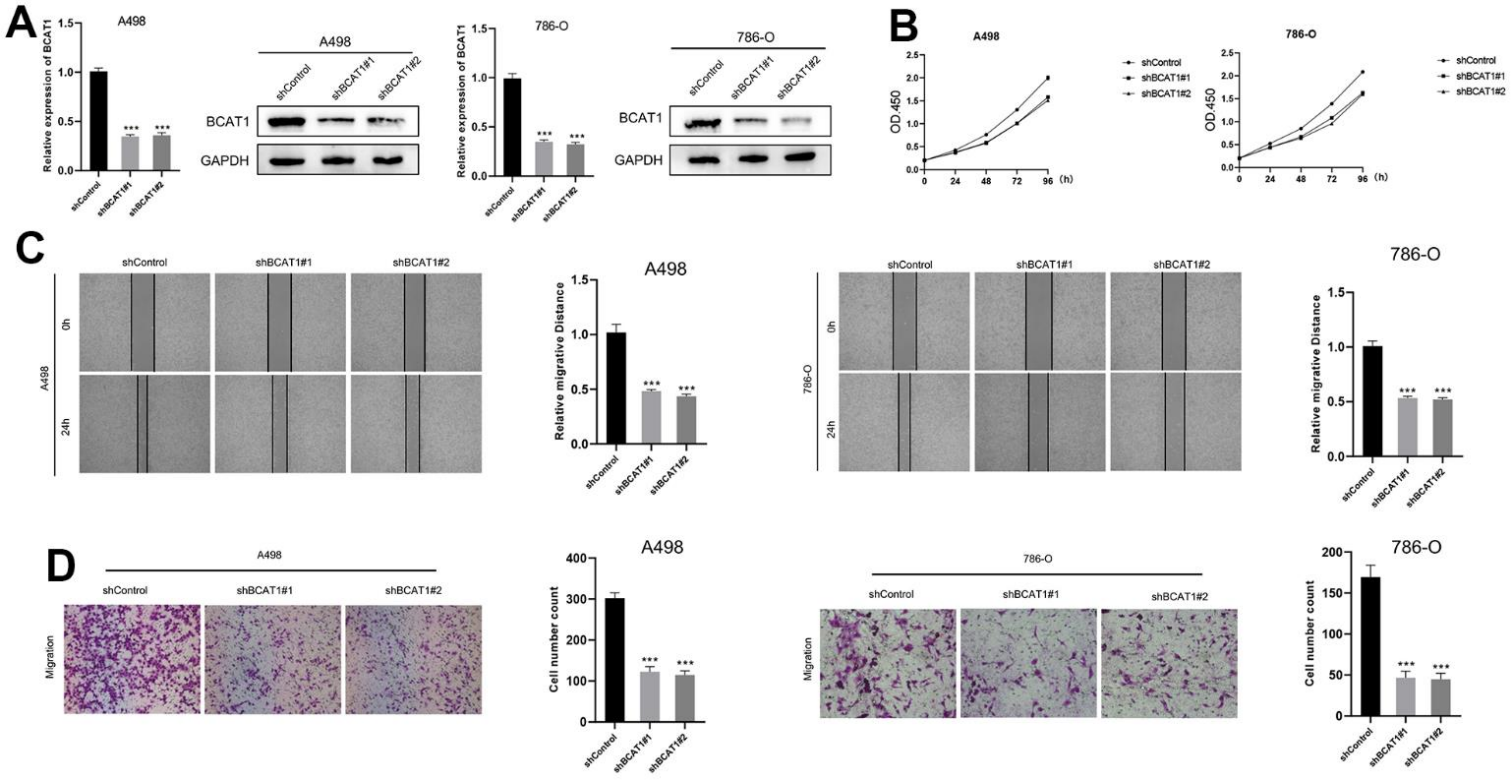
**Down-regulation of BCAT1 suppressed the progression of ccRCC *in vitro*.** (**A**) The expression of BCAT1 in A498 and 786-O cells was detected by RT-qPCR and Western blot; (**B**) BCAT1-knockdown suppressed ccRCC cell proliferation in A498 and 786-O cells; (**C**) Wound-healing tests demonstrated changes in ccRCC cell migration; (**D**) BCAT1-knockdown suppressed ccRCC cell metastasis in A498 and 786-O cells.

In the BCAT1-knockdown group, mRNA, and protein expression of BCAT1 were dramatically down-regulated (Figure 9A). The CCK8 assay demonstrated that the proliferation of A498 and 786-O cells was markedly decreased in BCAT1-knockdown group (Figure 9B). Wound healing detection suggested that the healing distance of A498 and 786-O cells in BCAT1-knockdown group was lower than that in control group after 24 hours (Figure 9C). Transwell experiments revealed that the migration of A498 and 786-O cells were clearly inhibited in BCAT1-knockdown group (Figure 9D). Therefore, the expression of BCAT1 was positively correlated with the proliferation, migration, and invasion of ccRCC cells.

## DISCUSSION

ccRCC is an immune-responsive tumor characterized by high heterogeneity and metastatic potential [23]. Despite the efficacy of combining molecular targeted drugs (Sunitinib and Pazopanib) with immune checkpoint blockade (Nivolumab) in improving the prognosis of advanced RCC patients, the incidence of ccRCC continues to rise annually [25]. In-depth investigations into ccRCC reveal that traditional histopathological features (tumor size, stage, grade) may not sufficiently address its diagnosis and prognosis [26–28]. A better understanding and integration of the latest advancements in deep learning and artificial intelligence technologies related to data processing and analysis are expected to assist in the diagnosis and prevention of diseases [29, 30]. BCAAs play a pivotal role in tumor proliferation and progression due to the heightened demand for BCAAs driven by the inherent dividing and proliferative properties of tumor cells [26]. Consequently, our study delves into the prognostic and immune characteristics of BMGs in ccRCC based on their expression profiles.

In our investigation, two BCAA metabolism-related clusters were developed, revealing distinct prognostic and immune features based on the expression profiles of 9 prognostic BMGs. Patients in cluster B exhibited a poor prognosis, a high abundance of immunosuppressive cells, and expression of immune checkpoint molecules. This phenomenon may be attributed, in part, to the elevated expression of BCAA transporter genes in cluster B, providing additional building blocks for tumor proliferation. Furthermore, significant enrichment of pro-cancer pathways (P53 signaling pathway) and epigenetic processes (cell cycle, DNA replication, and mismatch repair) in Cluster B potentially offers additional pathways for tumor progression [31]. Subsequently, the LASSO algorithm was employed to construct the BCAA metabolic prognostic signature and the formula for its risk score, denoted as BMS. The stability of this model in predicting patient prognosis was validated through univariate and multivariate independent prognostic analyses using datasets from E-MATB-1980 and GSE22541. In TME, invasive immune cells play a pivotal role in tumor proliferation, metastasis, and regulation of anti-cancer immunity, presenting critical therapeutic targets [18]. In the high-BMS group exhibited higher TME scores and increased immunosuppressive cell infiltration, correlating with a worse prognosis. This observation aligns with the immune exhaustion phenotype reported in Meng et al.’s study [32]. While immune checkpoint blocking therapy and targeted therapy are indispensable in the management of ccRCC [33], formulating an optimal personalized treatment plan remains a challenge for clinicians. Considering CTLA4 and PD-1 as key immune checkpoints, we analyzed differences in responses to immunotherapy between high- and low-BMS groups. The findings revealed that the effect of CTLA4(+) and/or PD-L1(+) treatment was superior in the high-BMS group. Additionally, patients in the high-BMS group exhibited increased sensitivity to four drugs (Rapamycin, Temsirolimus, Sorafenib, and Sunitinib). These results suggest that our BCAA metabolic prognostic model serves as a valuable indicator for predicting the efficacy of targeted therapy and immunotherapy, facilitating the development of individualized treatment for ccRCC patients.

Several physiological and pathological processes, encompassing tumour growth and metastasis, cell cycle, apoptosis, pyroptosis, and angiogenesis, are under the regulatory influence of BCAT1-mediated BCAA catabolism [34, 35]. It is well-established that distinct modes of cell death in ccRCC exhibit substantial heterogeneity across various dimensions, including functional status, tumor microenvironment, genomic alterations, responses to chemotherapy and immunotherapy, as well as clinical outcomes [36]. Numerous studies have established a correlation between elevated BCAT1 expression in tissues and the development of various cancers, such as glioblastoma [37], radiation-induced breast cancer [38], acute myeloid leukemia [16], and gastric cancer [39]. Our findings affirm a positive association between BCAT1 expression levels and poor prognosis in ccRCC. Gene Set Enrichment Analysis (GSEA) highlights the potential impact of BCAT1 on pathways related to tumor invasion and proliferation, including cytokine-cytokine receptor interaction, ECM receptor interaction, TGF-β signaling pathway, and JAK/STAT signaling pathway. These pathways are known to significantly influence tumor genesis and metastasis.

Furthermore, our GSEA enrichment analysis indicates that several crucial angiogenesis pathways, such as Toll-like receptor signaling, VEGF signaling pathway, and mTOR signaling, are markedly enriched in the high-BCAT1 group. Recent reports substantiate the close relationship between BCAT1 expression and mTOR signaling activity in other cancers. For instance, BCAT1, by enhancing mitochondrial biosynthesis and function through mTOR mediation, may stimulate breast cancer cell growth [38]. Activation of the PI3K/Akt/mTOR pathway by BCAT1 may also promote the proliferation, invasion, and angiogenesis of gastric cancer cells [40]. The PI3K/Akt/mTOR signaling pathway could induce the secretion of vascular endothelial growth factors (VEGFs) mediated by the hypoxia-inducible factor 1 (HIF-1) and get involved in regulating the expression of other angiogenic factors such as nitric oxide and angiopoietin [41]. In addition, the Toll-like receptor signaling pathway could also promote the angiogenesis of tumors by inducing VEGF production [42]. Given that BCAAs, especially leucine, serve as vital signaling molecules in the PI3K/AKT/mTOR pathway and can activate it in conjunction with growth factors [43, 44], our hypothesis posits that high BCAT1 expression may activate the PI3K/Akt/mTOR pathway via the production of essential BCAAs, consequently enhancing ccRCC angiogenesis.

Moreover, BCAT1 expression significantly correlates with immunosuppressive cells, particularly macrophages, and immunosuppressive checkpoints, potentially contributing to the formation of the immunosuppressive TME. Lidia et al. reported BCAT1 overexpression in glioblastoma, where it participates in constructing an immunosuppressive microenvironment by inhibiting the phagocytosis of tumor-associated macrophages [45]. These findings underscore BCAT1 as a promising molecular target for innovative ccRCC prevention and treatment strategies.

However, our current efforts are not without limitations. Firstly, our study relies solely on public data for constructing and retrospectively validating our findings. Thus, prospective studies are imperative to assess clinical utility in ccRCC patients. Additionally, comprehensive experiments are indispensable for elucidating the intricate mechanism of BCAA metabolism and BCAT1 in ccRCC.

## CONCLUSIONS

In summary, based on the expression of prognosis-related BMGs in ccRCC, we categorized patients into two BCAA metabolism-related clusters, each exhibiting distinct prognosis and immune cell infiltration characteristics. Additionally, we developed a BCAA metabolic prognostic signature for ccRCC, serving as a reliable predictor for both prognosis and immunotherapy response. Concurrently, our findings suggest that BCAT1 potentially contributes to the establishment of an immunosuppressive TME in ccRCC, positioning it as a promising therapeutic target.

## Supplementary Material

Supplementary Figures
