# Supplementary material for: Integrated single-cell and bulk characterization of branched chain amino acid metabolism-related key gene BCAT1 and association with prognosis and immunogenicity of clear cell renal cell carcinoma
Source: Aging (Albany NY). 2024 Feb 2;16(3):2715–35. doi: 10.18632/aging.205506 (PMC10911380; doi:10.18632/aging.205506)
Supplement: Supplementary Figures [file aging-16-205506-s001.pdf]

SUPPLEMENTARY FIGURES

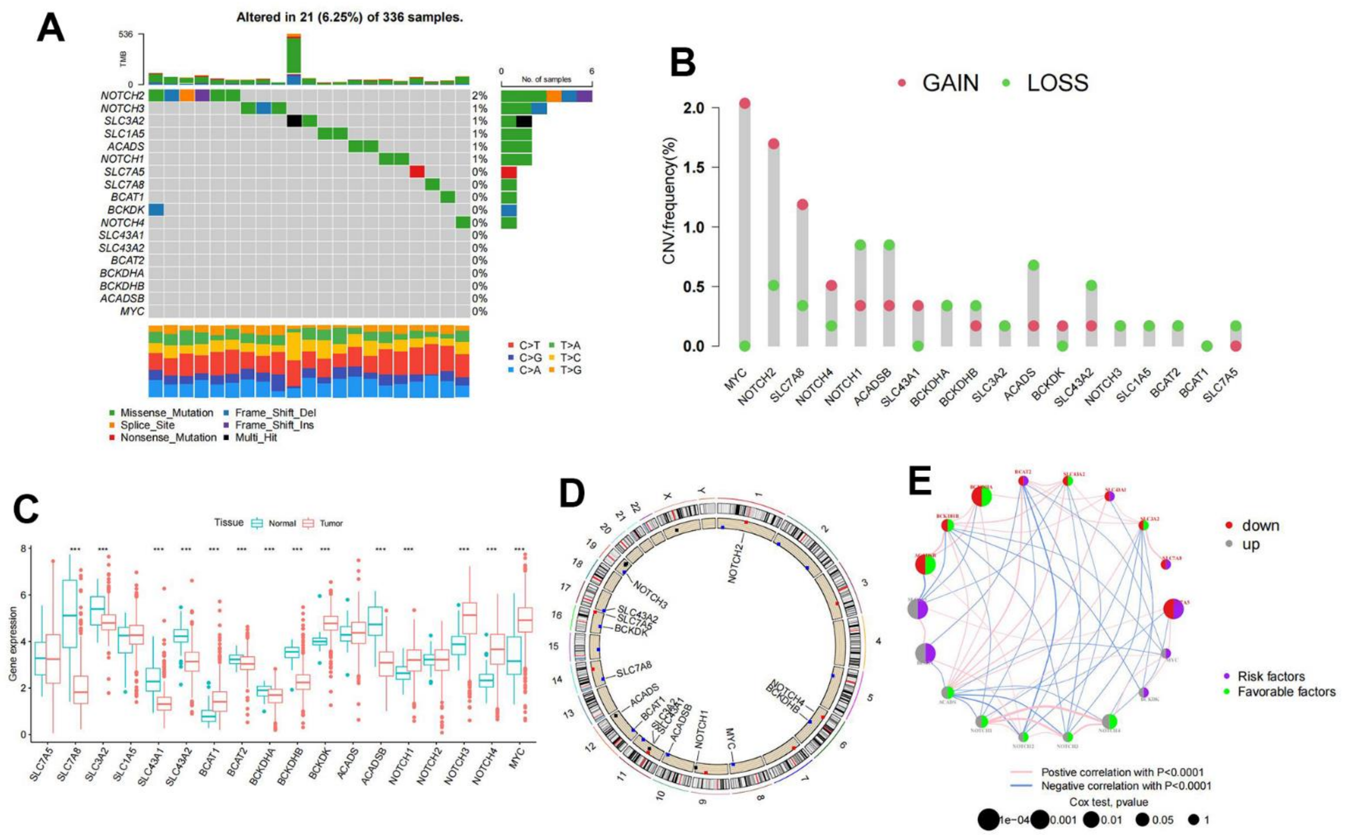

Supplementary Figure 1. Multi-omics landscape of BMGs in ccRCC. (**A**) Mutation frequency of 18 BMGs in 336 ccRCC patients. (**B**) Copy number variations (CNVs) of the 18 BMGs. (**C**) Differential expression of 18 BMGs in ccRCC and adjacent normal tissues. (**D**) Location of CNV alterations in the 23 chromosomes of 18 BMGs. (**E**) Association and prognostic characteristics of 18 BMGs in ccRCC. Wilcox test was used, and the asterisks represent the statistical *P*-value (**p* <0.05, ** *p* <0.01, *** *p* <0.001).

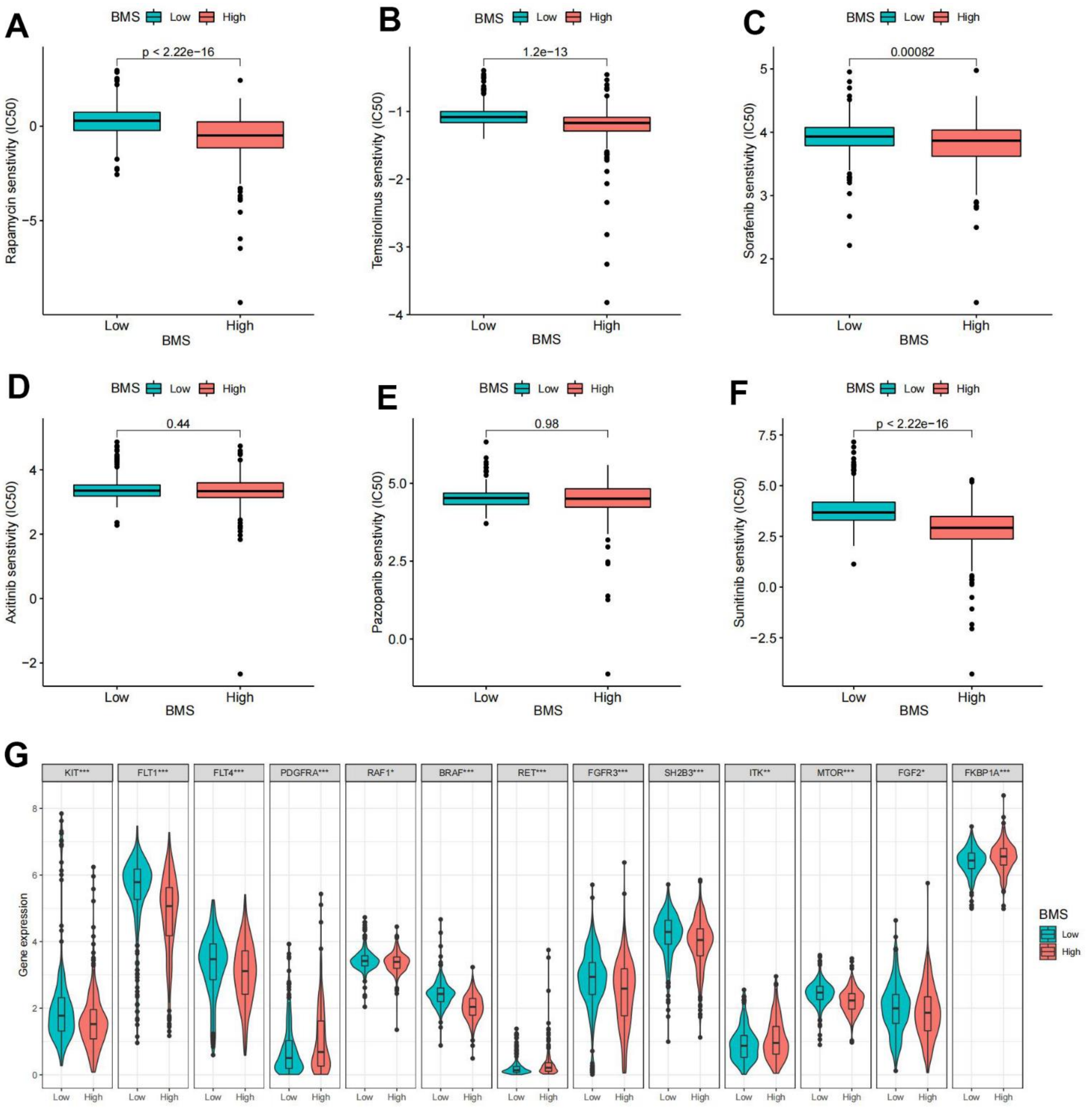

Supplementary Figure 2. Drug sensitivity analysis of BMS groups. (**A**–**F**) Comparison of the sensitivity of patients in high- and low-BMS groups to (**A**) Rapamycin, (**B**) Temsirolimus, (**C**) Sorafenib, (**D**) Axitinibn, (**E**) Pazopanib, and (**F**) Sunitinib. (**G**) Differential expression of target genes between high- and low-BMS groups after targeted drug treatment. Wilcox test was used, and the asterisks represent the statistical *P*-value (**p* <0.05, ** *p* <0.01, *** *p* <0.001).

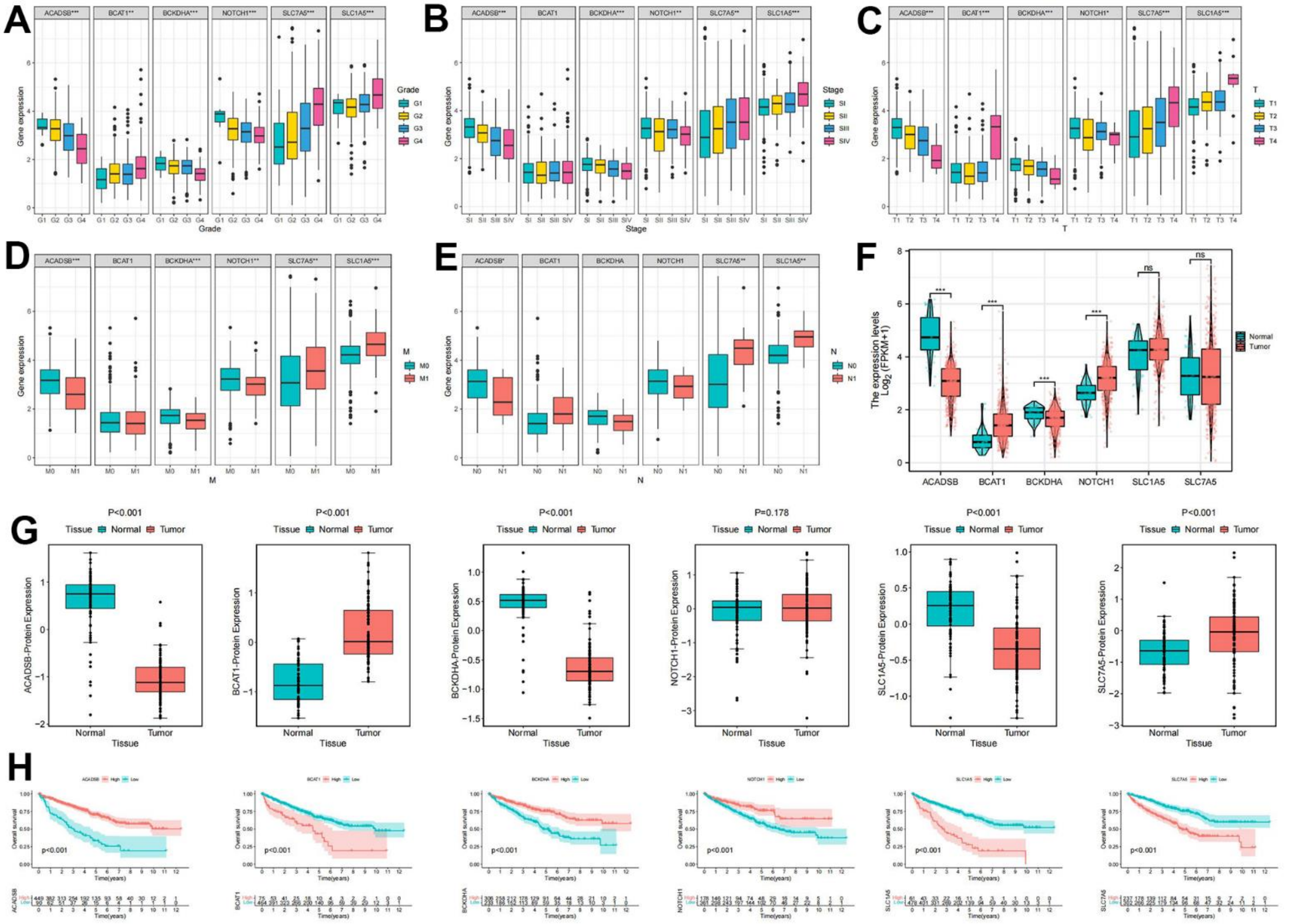

Supplementary Figure 3. Clinical, prognostic, and protein expression characteristics of model genes. (**A**–**E**) Differential expression of model genes among different clinicopathological variables, including (**A**) histological grade, (**B**) pathological stage, (**C**) T stage, (**D**) M stage, and (**E**) N stage. (**F**) Differential mRNA expression of model genes between ccRCC and adjacent tissues. (**G**) Differential protein expression of model genes between ccRCC and adjacent tissues. (**H**) Survival analysis of model genes in ccRCC. Wilcox test was used, and the asterisks represent the statistical *P*-value (**p* <0.05, ** *p* <0.01, *** *p* <0.001).
